# Transcriptional regulation of ependymal cell maturation within the postnatal brain

**DOI:** 10.1186/s13064-018-0099-4

**Published:** 2018-02-16

**Authors:** Diana Vidovic, Raul Ayala Davila, Richard M. Gronostajski, Tracey J. Harvey, Michael Piper

**Affiliations:** 1 The School of Biomedical Sciences, The University of Queensland, Brisbane, QLD 4072 Australia; 2 Department of Biochemistry, Program in Genetics, Genomics and Bioinformatics, Center of Excellence in Bioinformatics and Life Sciences, State University of New York at Buffalo, Buffalo, New York 14260 USA; 3 Queensland Brain Institute, The University of Queensland, Brisbane, 4072 Australia

**Keywords:** Ependymal cell, NFIX, Transcription factor, Cerebral cortex, Radial glia

## Abstract

**Background:**

Radial glial stem cells within the developing nervous system generate a variety of post-mitotic cells, including neurons and glial cells, as well as the specialised multi-ciliated cells that line the walls of the ventricular system, the ependymal cells. Ependymal cells separate the brain parenchyma from the cerebrospinal fluid and mediate osmotic regulation, the flow of cerebrospinal fluid, and the subsequent dispersion of signalling molecules via the co-ordinated beating of their cilia. Deficits to ependymal cell development and function have been implicated in the formation of hydrocephalus, but the transcriptional mechanisms underpinning ependymal development remain poorly characterised.

**Findings:**

Here, we demonstrate that the transcription factor nuclear factor IX (NFIX) plays a central role in the development of the ependymal cell layer of the lateral ventricles. Expression of ependymal cell-specific markers is delayed in the absence of *Nfix*. Moreover, *Nfix-*deficient mice exhibit aberrant ependymal cell morphology at postnatal day 15, culminating in abnormal thickening and intermittent loss of this cell layer. Finally, we reveal *Foxj1*, a key factor promoting ependymal cell maturation, as a target for NFIX-mediated transcriptional activation.

**Conclusions:**

Collectively, our data indicate that ependymal cell development is reliant, at least in part, on NFIX expression, further implicating this transcription factor as a mediator of multiple aspects of radial glial biology during corticogenesis.

**Electronic supplementary material:**

The online version of this article (10.1186/s13064-018-0099-4) contains supplementary material, which is available to authorized users.

## Introduction

Ependymal cells play a central role in many aspects of central nervous system biology. These epithelial cells line the ventricular cavities of the brain and spinal cord, and act as a physical barrier between the ventricular cerebrospinal fluid and the brain parenchyma [15]. Ependymal cells contain both adherens junctions and tight junctions, and their expression of transporters enables the osmolarity of the cerebrospinal fluid to be tightly regulated via the uptake of ions and water molecules [27]. Ependymal cells also project multiple cilia into the ventricular cavities. The co-ordinated beating of these motile cilia is required to facilitate bulk flow of cerebrospinal fluid from the lateral ventricles towards the third and fourth ventricles, and then towards the subarachnoid space, where it is absorbed [38, 39]. This function is vital to ensure that the continual production of cerebrospinal fluid by the choroid plexuses is balanced by its reabsorption into the venous system. Ependymal cells have also been implicated in providing trophic support and signalling molecules to underlying neural tissues. For example, ependymal cells produce fibroblast growth factors [6] and vascular endothelial growth factor [2], and hence support growth and vascular system development within the embryonic brain. Finally, ependymal cells have been implicated in regulating aspects of neurogenesis within the adult forebrain, including neural stem cell self-renewal [33], neurogenesis [18] and neuroblast migration [16]. The importance of ependymal cells is further highlighted by the fact that deficits in ependymal cell differentiation, maturation and function are linked to a severe neurological disorder known as hydrocephalus, which is defined by the excessive accumulation of cerebrospinal fluid within the ventricular system [24].

Ependymal cells are derived from the neural stem cells of the developing nervous system, the radial glia [35]. The differentiation of radial glial first produces neurons, then glial cells such as astrocytes, and finally ependymal cells. In rodents, ependymal cell differentiation mostly occurs in late gestation, and the maturation of these cells predominantly occurs during the first two weeks after birth [35]. Although we now know a great deal about the molecular cascades that regulate neuronal differentiation, migration and maturation [8, 11], our understanding of the factors that promote ependymal cell development is far more limited. At the transcriptional level, the forkhead transcription factor FOXJ1 has been shown to be essential for ependymal cell maturation and ciliogenesis [14, 40]. Similarly, the homeobox factor SIX3 has also been implicated in ependymal cell maturation, as well as the suppression of genes involved in radial glial maintenance [17].

Recent studies have demonstrated that the transcription factor nuclear factor IX is expressed by mature ependymal cells [4, 12], and that *Nfix*-deficient mice exhibit communicating hydrocephalus defined by extensive expansion of the lateral ventricles [3, 36]. NFIX is one of four proteins in the NFI family, and these transcription factors have been shown to regulate a variety of cellular processes, including differentiation [23, 28], migration [37] and quiescence [20]. Within the nervous system, *Nfix*^*−/−*^ mice have been shown to exhibit deficits in the development of the neocortex, spinal cord, hippocampus and cerebellum [5, 10, 12, 13, 23, 29]. At a molecular level, NFIX has been shown to promote radial glial differentiation via the expression of lineage-specific genes including *Gfap* [7], whilst repressing genes important for radial glial self-renewal, including *Sox9* [12]. In *Nfix*^*−/−*^ mice, hydrocephalus becomes fully penetrant in the early postnatal period [36], but the contribution of NFIX to ependymal cell development remains unclear. Here, we report that ependymal cell maturation is delayed in the absence of *Nfix*, and that *Foxj1* is a target for transcriptional activation by NFIX within the developing brain. Collectively, these findings illustrate a novel role for the NFI family in mediating the maturation of a key cellular subtype, the ependymal cell, during nervous system development.

## Methods

### Animal ethics

The work performed in this study conformed to The University of Queensland Animal Welfare Unit guidelines for animal use in research (AEC approval numbers QBI/353/13/NHMRC and QBI/383/16). Experiments were performed in accordance with the Australian Code of Practice for the Care and Use of Animals for Scientific Purposes, and were carried out with approval from The University of Queensland Institutional Biosafety Committee.

### Animals

*Nfix*^*+/+*^ and *Nfix*^*−/−*^ mice were maintained on a C57Bl/6 J background. The *Nfix* allele was initially generated as a conditional line [3]. The *Nfix* targeting vector was constructed with a 4.2 kb 5′ homology arm containing all of exon 2 and 633 base pairs of intron 2 [3]. The knockout allele was generated through cre-mediated recombination, using a strain under which cre recombinase expression was controlled by the promoter from the *ZP3* promoter, which is expressed in the developing oocyte prior to the first meiotic division, ensuring germline deletion of the targeted gene [3]. Once the *Nfix* knockout allele was generated, this strain was maintained on a C57Bl/6 J background. *Nfix*^*+/−*^ sires were placed with *Nfix*^*+/−*^ dams to obtain litters containing *Nfix*^*+/+*^*, Nfix*^*+/−*^, and *Nfix*^*−/−*^ mice, which were generated at the expected Mendelian ratios. Embryos were genotyped by polymerase chain reaction (PCR) [3].

### Preparation of fixed brain tissue

Postnatal pups from postnatal day (P) 0 to P15 were transcardially perfused with 0.9% saline, followed by 4% paraformaldehyde. The skull of the animals were removed and the brains were post fixed for 1 week, then stored at 4 °C in phosphate buffered saline (PBS) until required. Brains were embedded in noble agar and sectioned in the coronal plane at 50 μm using a vibratome (Leica, Deerfield, IL). Exceptions to this protocol were sections for the iDISCO protocol, in which sectioning was performed at a thickness of 200 μm.

### Antibodies and immunofluorescence

Sections were mounted on slides before heat-mediated antigen retrieval was performed in 10 mM sodium-citrate solution 95 °C for 15 min. Immunofluorescence staining was performed as described previously [4]. Briefly, sections were covered in a blocking solution containing 2% normal serum and 0.2% Triton-X-100 for 2 h. Primary antibodies were diluted in blocking solution and were incubated with the sections overnight at 4 °C. The following day, fluorescently conjugated secondary antibodies diluted in block were incubated with the sections for 2 h. Sections were then counterstained with 4′,6-diamidino-2-phenylindole (DAPI) (Invitrogen, Carlsbad, CA) and cover-slipped using DAKO fluorescent mounting media. Details of the antibodies used in the study can be found in Tables 1 and 2.

**Table 1:** Primary antibodies used in this study

Antibody	Host	Source	Dilution
Acetylated tubulin	Mouse monoclonal	Sigma-Aldrich, T7451	1/1000
FOXJ1	Mouse monoclonal	eBioscience	1/1000
N-cadherin	Rabbit polyclonal	Abcam, ab18203	1/200
NFIX	Rabbit polyclonal	Abcam, ab101341	1/100
NFIX	Mouse monoclonal	Sigma-Aldrich, SAB1401263	1/200
s100β	Mouse monoclonal	Sapphire Bioscience, ab66028	1/400
Vimentin	Rabbit polyclonal	Abcam, ab92547	1/500

**Table 2:** Secondary antibodies used in this study

Secondary Antibodies	Company	Dilution
Goat-anti-rabbit Alexa Fluor 488	Invitrogen	1/125
Donkey-anti-mouse Alexa Fluor 647	Invitrogen	1/250
Donkey-anti-rabbit Alexa Fluor 568	Invitrogen	1/250

### iDISCO+ staining and clearing

Fixed samples were dehydrated with a methanol series, then placed in a solution of 66% dichloromethane (DCM)/33% methanol at room temperature for 1 h and then washed twice in 100% methanol for 1 h. Samples were treated with 5% hydrogen peroxide in methanol overnight at 4 °C. Samples were then rehydrated with a methanol/H_2_O series, then washed twice in PBS containing 0.2% TritonX-100 for 1 h. For immunolabelling, samples were incubated overnight in a solution containing 0.2% Triton X-100, 20% dimethyl sulfoxide (DMSO), 0.3 M glycine in PBS at 37 °C overnight, then blocked in 0.2% Triton X-100, 10% DMSO, 6% donkey serum in PBS at 37 °C for 2 days. Samples were incubated with primary antibody for seven days at 37 °C in blocking solution (0.2% Tween-20, 10 μg/ml heparin, 5% DMSO and 3% donkey serum diluted in PBS). The following day samples were rinsed in blocking solution, before being incubated with secondary antibody diluted in blocking solution for three days at 37 °C. Samples were washed again, then cleared. To do this, samples were dehydrated with a methanol series, followed by an over-night incubation in 66% DCM/33% methanol at room temperature. Samples were then washed twice in 100% DCM for 15 min each. Finally, samples were rehydrated with in a methanol series, and cover slipped using DAKO fluorescent mounting media.

### Imaging acquisition

Confocal images were acquired as consecutive 1 μm optical sections spanning a 10 μm thick Z-stack on a Zeiss inverted Axio-Observer fitted with a W1 Yokogawa spinning disk module and Hamamatsu Flash4.0 sCMOS camera and Slidebook software (3i) [26]. Exceptions to this protocol were sections for the iDISCO protocol, in which images were acquired at a thickness 0.165 μm and optical sections spanning a 100 μm to 150 μm thick Z-stack.

### Image deconvolution and processing

To obtain well-sampled image stacks that could be processed with deconvolution images were sampled at a rate close to the ideal Nyquist rate. The Nyquist sampling distance in the lateral direction was calculated as: Δx = Δy = λex4kn × sinα and for the axial direction, the Nyquist sampling distance was calculated as: Δz = λex2kn × (1 − cosα), where *n* is the lens medium refractive index (1.338 for water), *k* is the number of excitation photons (photon count; set to 2 for MPE microscopy), *λ*ex is the wavelength of the excitation light, and *α* is the half-aperture angle of the objective and the focal plane interval (*Z*) was set to 0.165 μm, sufficient to satisfy Nyquist rate sampling according to the stated equations. Huygens Essential (version 4 64-bit, Scientific Volume Imaging, Hilversum, The Netherlands) was used to remove noise and reassign out-of-focus light with a theoretically calculated point spread function, using the classic maximum likelihood estimation (CMLE) deconvolution algorithm. In addition, the object stabilizer module of Huygens Essential was used to align images along the *Z-*axis to compensate for drift and other mechanical instabilities. Processed image stacks were saved in 16-bit TIFF format, utilizing the whole dynamic range [41]. Multichannel simulated fluorescence projection images were generated by using the IMARIS software package (Bit- plane AG, Zurich, Switzerland) and were further processed for display by using PhotoShop software (Adobe). Subtraction of the background from the images was performed with the IMARIS software package to remove the host tissue autofluorescence. Images were then rotated to be viewed in the Z plane.

### Chromatin immunoprecipitation (ChIP)-qPCR

SVZ tissue from P15 wild-type mice was dissociated, then fixed in 1% formaldehyde for 8 min. Nuclei were lysed and chromatin sonicated using 14 cycles (30 s on/30 s off) of the Bioruptor Pico (Diagenode, Belgium) so that the majority of chromatin was between 100 and 500 base pairs in length. Immunoprecipitation was performed with 8 μg of rabbit anti-NFIX (Sigma-Aldrich, SAB1401263) or 8 μg of rabbit IgG control antibody coupled to 40 μl of Protein G Dynabeads (10003D, Thermo Fisher Scientific). DNA purification was performed using a Qiagen PCR purification kit. Isolated DNA was quantified using qPCR as described previously [28]. We designed two primer sets, to encompass the − 935 and − 2856 NFI motifs, respectively. These were: − 935 Forward: 5’ GTCAGTGGCTCAGGCTCTT, Reverse: 5’ GGCAGATGCAGAAGCAGGTA. -2856 Forward: 5’ ACGCCTAAGGGAGAGGGTAG, Reverse: 5’ GAGGTGCAGGAGACCAACTC. Enrichment of NFIX occupancy at the *Foxj1* promoter relative to the IgG control was calculated as a percentage of total input.

### Reporter gene assay

The NFI binding motif was derived from a previously published chromatin immunoprecipitation-sequencing (ChIP-seq) dataset generated with a pan-NFI antibody [30]. Potential NFI binding sites were identified using the MEME algorithm and the FIMO motif-scanning program as described previously [12]. FIMO was run on the mouse genome (without repeat masking) using a 0-order background generated on the entire mouse genome, and a pseudocount of 0.1. All potential binding sites with *p*-value ≤10^− 4^ were reported in the region of − 3000 base pairs to + 200 base pairs relative to the transcription start site (TSS) of relevant genes. Putative NFI binding sites near the *Foxj1* promoter were identified by viewing the FIMO output using the UCSC genome browser. Our bioinformatics promoter screen identified two potential NFI binding sites within the *Foxj1* promoter (uc007mkv.2). One was located at − 2856 (chromosome 10: 58,146,618–58,146,632 TTGGTTGGAAGCCAG), base pairs relative to the TSS and the other at − 935 (chromosome 10: 58,146,618–58,146,632 TGGGTACAAGGCCAG) base pairs relative to the TSS. We generated a luciferase construct containing a 1077 base pair fragment of the mouse *Foxj1* promoter region, which contained the most proximal binding motif relative to the TSS (− 935). This was generated using the following primers: forward 5’-ATTCGTTTGTGAACGTATTCG-3; reverse 5′- CGGTTCTCTGTCCCCAGCT-3′. The amplicon was inserted into the MluI and BglII restriction enzyme sites and cloned upstream of the Renilla luciferase gene pLightSwitch_Prom vector (Switchgear Genomics; *Foxj1 pLuc*). The vectors used in the luciferase assay were a full-length *Nfix* cDNA construct driven by a CMV early enhancer/ chick β-actin promoter and splice acceptor rabbit β-globulin (*NFIX pCAGIG*) or an empty vector control (*pCAGIG*) [22]. Neuro 2A cells were seeded 1*10^4^ cells per well 24 h prior to transfection. DNA was transfected using Lipofectamine 2000 (Thermo Fisher). *Cypridina* (Switchgear Genomics) was added as an internal control for transfection efficiency [28]. Luciferase activity was measured at 24 h post-transfection using a dual-luciferase system (Switchgear Genomics). Within each experiment, each treatment was replicated three times. Each experiment was also independently replicated three times.

### Statistical analyses

The parameters of our statistical testing approach were specified prior to data collection. Two-tailed unpaired Students *t-*tests were performed when comparing two groups. ANOVA was performed when comparing more than two groups. Error bars represent the standard error of the mean.

## Results and discussion

**Fig. 1:**
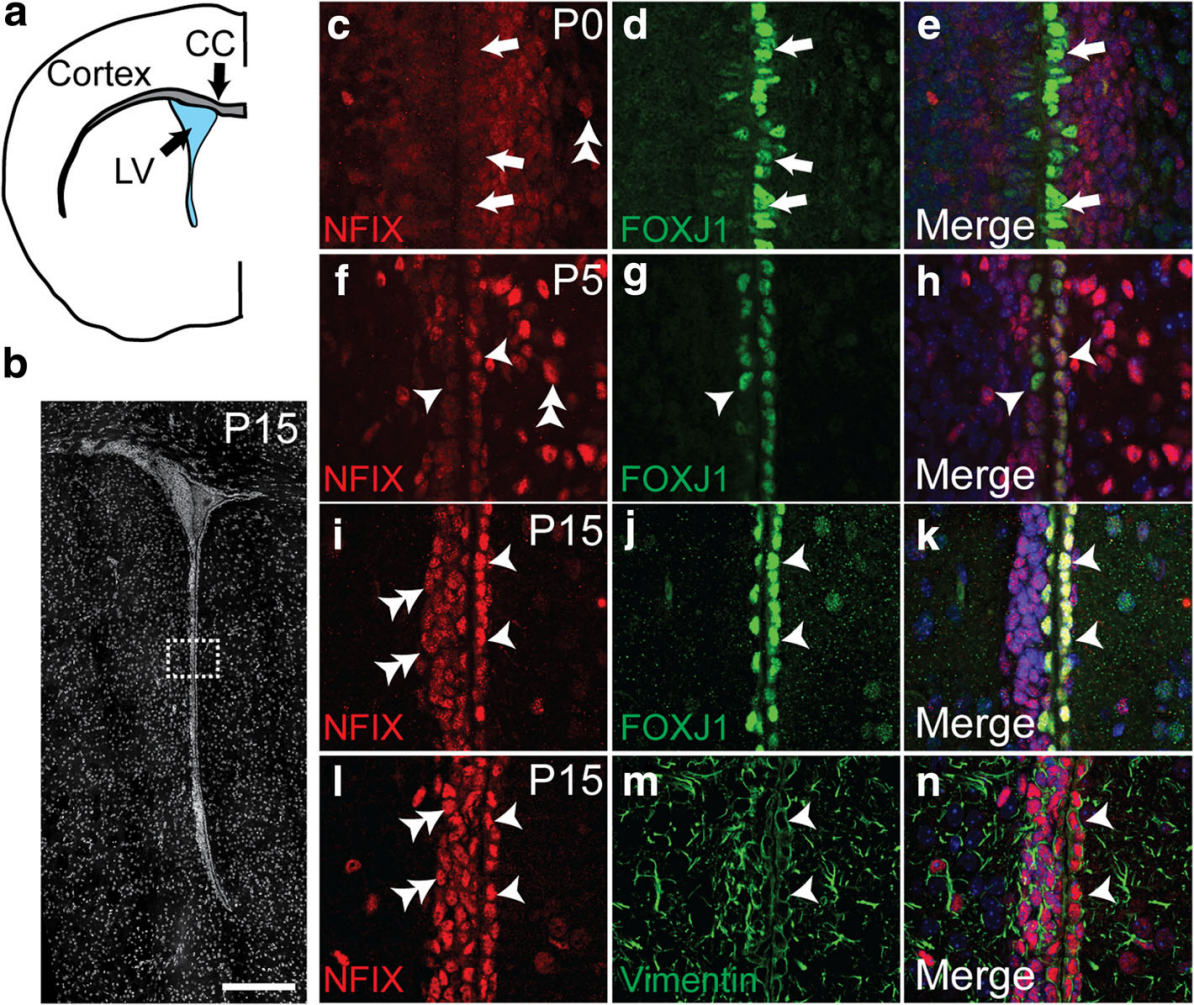
NFIX is expressed by maturing ependymal cells lining the lateral ventricles. **a** Schematic of a coronal section of a postnatal rodent cerebral cortex, at the level of the corpus callosum (CC). LV = lateral ventricle. **b** Coronal section of a P15 mouse brain stained with DAPI, showing one of the lateral ventricles. The boxed region depicts the approximate region at which imaging was performed. **c**-**e** Higher power view of the lateral ventricle region of a P0 brain, showing the expression of NFIX (red, **c**) and FOXJ1 (green, **d**). Whereas FOXJ1 expression is evident in ependymal cells at this age, these cells only expressed low levels of NFIX (arrows in **c**-**e**). At P5 and P15, co-expression of NFIX and FOXJ1 is clearly evident within ependymal cells (arrowheads in **f**-**h** and **i**-**k**). This was confirmed with the use of a second ependymal cell marker, vimentin, at P15, as NFIX-immunopositive nuclei were clearly surrounded by vimentin-positive filaments (arrowheads in **l**-**n**). At all of these ages, NFIX-positive cells were also detected within the developing ventricular-subventricular zone region (double arrowheads in **c**, **f**, **i**, **l**). Scale bar (in **b**): **b** 500 μm; **c**-**n** 30 μm

The expression of NFIX by radial glial cells within the developing and postnatal dorsal telencephalon has been previously documented [3], as has the expression of this factor within ependymal cells of the adult brain [4]. However, whether or not radial glia lining the walls of the lateral ventricles express NFIX during the period of postnatal development, when ependymal cells are maturing, is not clear. To address this, we performed co-immunofluorescence labelling, coupled with confocal microscopy, on samples at P0, P5 and P15, using antibodies against NFIX and the ependymal marker FOXJ1 [14]. To do this we used an anti-NFIX antibody whose specificity has previously been demonstrated [4] (Additional file 1). At P0, FOXJ1 expression was evident in developing ependymal cells of the lateral ventricle. NFIX was also expressed by these cells, albeit at a low level (Fig. 1a-e). By P5, however, the expression of NFIX by maturing ependymal cells was much more prominent, with all FOXJ1-expressing cells also clearly expressing NFIX (Fig. 1a, f-h). Similarly, at P15, all FOXJ1-expressing cells lining the lateral ventricles expressed NFIX (Fig. 1i-k). The expression of NFIX by ependymal cells at this age was further investigated via the use of another ependymal cell marker, namely vimentin [34]. This demonstrated that the cuboidal cells lining the ventricle labelled with vimentin possessed a strongly NFIX-immunoreactive nucleus (Fig. 1l-n). NFIX expression was also observed in cells adjacent to the ependymal cell layer, consistent with previous reports indicating that cells of the ventricular-subventricular zone neurogenic niche, and astrocytes, express this transcription factor [13, 31].

**Fig. 2:**
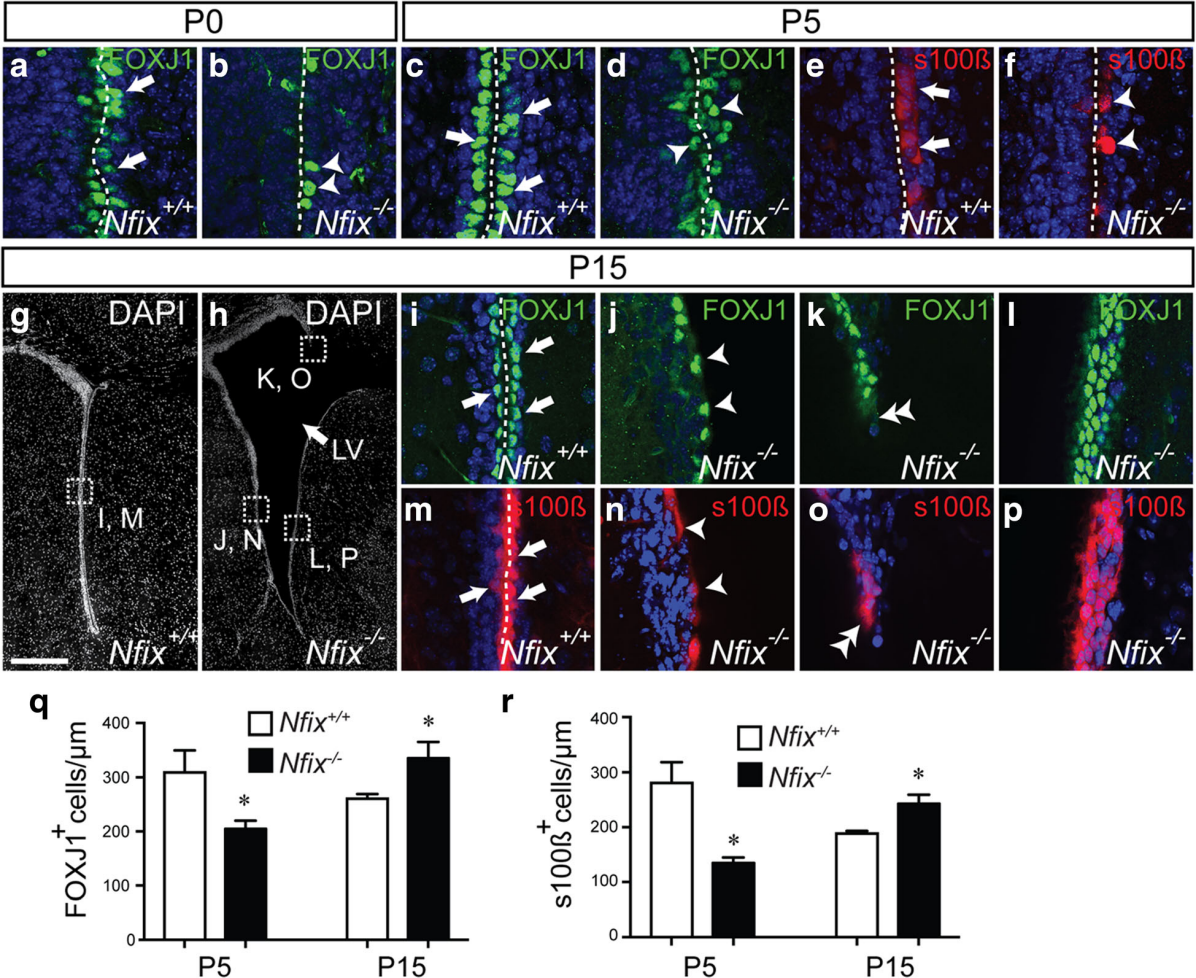
Ependymal cell maturation is delayed in the absence of *Nfix*. **a**-**p** Coronal sections of P0 (**a**, **b**), P5 (**c**-**f**) and P15 (**g**-**p**) mouse brains immunolabelled with antibodies against FOXJ1 (**a**-**d**, **i**-**l**) and s100β (**e**, **f**, **m**-**p**). The dotted lines indicate the ventricular wall. At P0 in the wild-type, FOXJ1 cells were seen lining the ventricles (arrows in **a**). In the mutant at this age, however, there were fewer of these cells (arrowheads in **b**). Similarly, at P5 there were fewer cells expressing either FOXJ1 (arrowheads in **d**) or s100β (arrowheads in **f**) in comparison to the controls (arrows in **c**, **e**). (**g**, **h**) Low magnification images of wild-type (**g**) and *Nfix*^*−/−*^ (**h**) P15 cerebral cortices labelled with DAPI, showing the areas from which the higher magnification images were taken. The lateral ventricle (LV) was significantly expanded in the mutant. In the wild-type, the ependymal cell layer was clearly labelled with either FOXJ1 or s100β (arrows in **i** and **m** respectively), revealing a uniform epithelial sheet. In the mutant, however, we observed regions in which there were reduced numbers of ependymal cells lining the ventricle (arrowheads in **j** and **n**), areas where the ependymal cell layer had sloughed off (double arrowheads in **k** and **o**), and areas in which the ependymal cell layer was more than one-cell thick (**l** and **p**). **q**, **r** Cell counts demonstrated that there were significantly fewer FOXJ1-expressing (**q**) and s100β-expressing (**r**) cells per unit length at P5 in the mutant, and, conversely, that there were significantly more of these cells in the mutant at P15 in comparison to the controls. * *p* < 0.05, *t*-test. Scale bar (in **g**): **g**, **h** 500 μm; **a**-**f**, **i**-**p** 30 μm

Given the expression of NFIX by ependymal cells during the early postnatal period when these cells are maturing, how does the absence of this transcription factor affect the development of this epithelial cell layer? To address this question, we analysed ependymal cell maturation in *Nfix*^*+/+*^ and *Nfix*^*−/−*^ mice over a range of postnatal ages. At P0, despite the low expression of NFIX at this age (Fig. 1c), there was already a marked difference in the expression of FOXJ1 between wild-type and mutant animals, with fewer cells expressing FOXJ1 in the mutant (Fig. 2a, b). Similarly, at P5 we saw significantly fewer cells expressing FOXJ1 in the mutant in comparison to the controls (Fig. 2c–q). A similar result was observed using a second marker for ependymal cells, s100β (Fig. 2e–r). By P15, a number of deficits were observed in the mutant, similar to preliminary reports [36]. The first observation was that *Nfix*^*−/−*^ mice at this age exhibited marked expansion of the lateral ventricles in comparison to the controls (Fig. 2g, h). At this age, the ependymal layer of the wild-type consisted of a coherent epithelial sheet, one cell thick, lining the ventricles. These cells strongly and specifically expressed both FOXJ1 and s100β (Fig. 2i, m). In the mutant, however, we observed three consistent deficits (Additional file 2). Firstly, where the ependymal cell layer appeared grossly intact, there were fewer ependymal cells present lining the ventricles (Fig. 2j, n). Secondly, we frequently observed areas in which the ependymal layer was discontinuous, consistent with these cells potentially sloughing away from the walls of the ventricle (Fig. 2k, o). Finally, we also observed areas in the mutant brain in which the lining of the ventricles exhibited aberrant thickening of the ependymal cell layer (Fig. 2l, p). Indeed, this thickening contributed to a statistically significant increase in number of FOXJ1-positive (Fig. 2g) and s100β-positive (Fig. 2r) cells per unit length of the mutant ventricle in comparison to the control at P15. Collectively, these data indicate that NFIX plays an important role in the formation of the mature ependymal layer within the lateral ventricles of the postnatal brain.

**Fig. 3:**
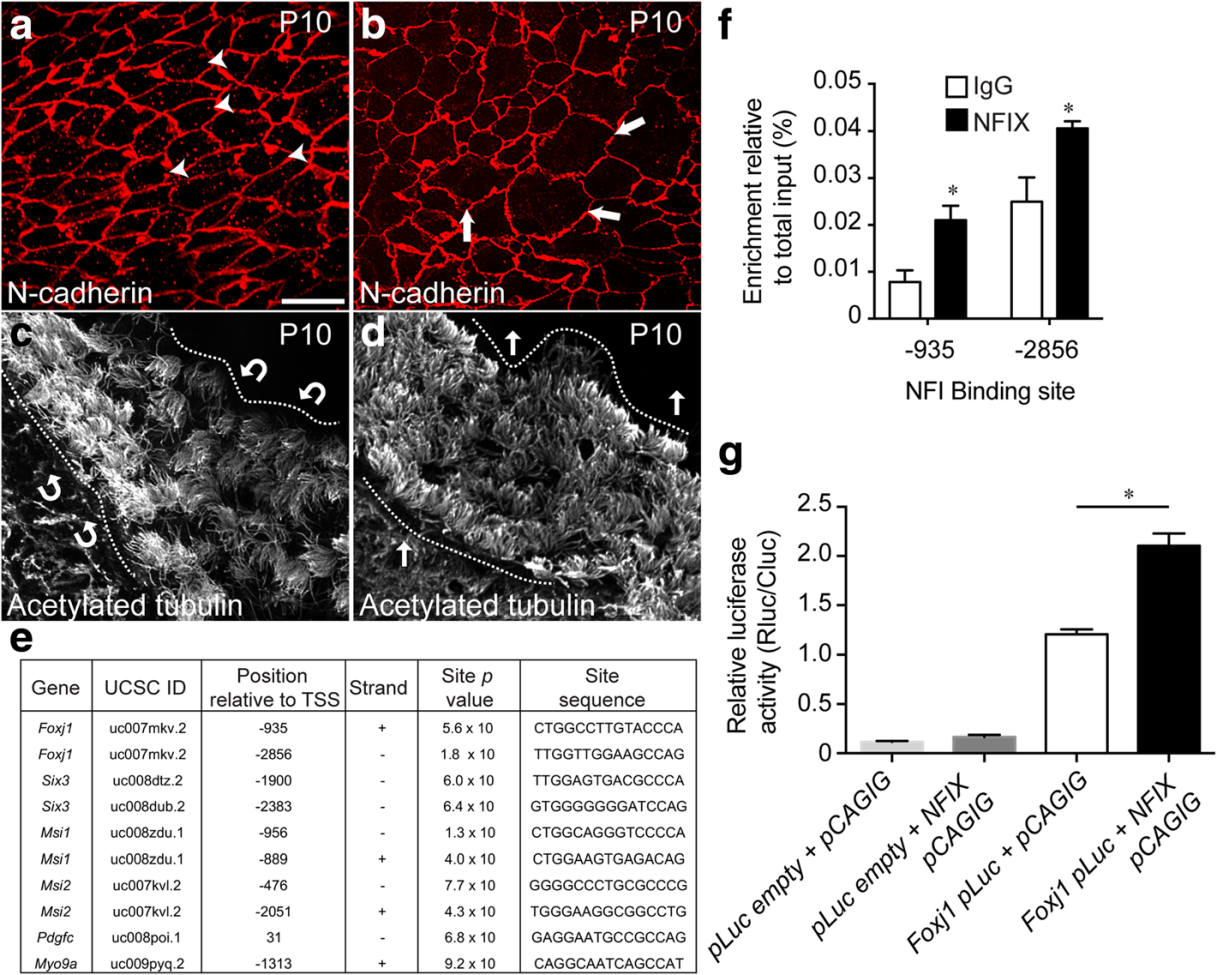
*Foxj1* is a target for transcriptional activation by NFIX. **a**-**b** *En face* view of the apical surface of wall of the lateral ventricle following immunostaining with N-cadherin (red) at P10 in control (**a**) and *Nfix*^*−/−*^ mutant (**b**) mice. In the control, N-cadherin labelling revealed the regular, cobblestone-like appearance of the ependymal epithelial sheet (arrowheads in **a**). In the mutant, however, ependymal cell shape was very irregular, and no coherent cobblestone-like array was observed (arrows in **b**). **c**, **d** *En face* imaging was also performed using antibodies against acetylated tubulin. In the control, wave-like patterns were clearly discernible in the cilia of the ependymal cells (arrows in **c** indicate the direction of bulk cilia movement). Conversely, in the mutant, ependymal cilia projected directly out into the ventricular lumen (**d**). **e** NFI binding motifs were identified in the basal promoters of numerous genes previously implicated in ependymal cell development using an in silico genome-wide screen. We identified two potential NFI binding sites within the *Foxj1* promoter. **f** ChIP-qPCR revealed significant enrichment of NFIX binding to the − 935 and − 2856 genomic sites in comparison to a non-specific antibody (IgG) in vivo. * *p* < 0.05, *t*-test. **g** A reporter gene assay revealed that NFIX was able to activate *luciferase* gene expression under the control of a fragment of the *Foxj1* promoter containing the − 935 site. * *p* < 0.05, ANOVA. Scale bar (in **a**): 20 μm

Ependymal cells form a continuous epithelial sheet that lines the ventricular surface. We next sought to address the structure of the remaining ependymal cell layer in mice lacking *Nfix*. To do this, we took wild-type and *Nfix*^*−/−*^ brains at P10, and performed N-cadherin staining, followed by clearing of the tissue using the iDISCO technique. We then performed confocal microscopy, taking a Z-stack through the apical surface of the ependymal sheet, followed by deconvolution and image rotation to enable an *en face* image of the apical surface of the ventricular wall to be viewed. For this analysis, we focussed on the regions of the mutant brain which had a partially continuous ependymal layer (Fig. 2j). In the wildtype, the N-cadherin clearly labelled the periphery of the cells, and revealed a regular cobblestone appearance of the ependymal epithelial sheet (Fig. 3a). In the mutant, however, although the expression of N-cadherin per se did not appear different from the control, the shape of the ependymal cells present was very irregular (Fig. 3b), a finding suggestive of potentially abnormal adherens junctional belt integrity. Next, we used the same technique to analyse the cilia of ependymal cells, using an antibody against acetylated tubulin. In the wild-type, the cilia observed were physically orientated in a pattern resembling co-ordinated beating (Fig. 3c). In the mutant, however, whereas cilia were clearly present, there appeared to be no co-ordinated pattern of ciliary direction, and most cilia were seen to be projecting directly out into the ventricular lumen (Fig. 3d). This further indicates that potential deficits in ependymal cell maturation could be evident in the absence of *Nfix*.

To identify a molecular mechanism by which NFIX promotes ependymal cell maturation, we mined a previously described in silico genome-wide screen for NFI binding sites [12], focussing on genes implicated in ependymal cell development. Interestingly, we identified potential NFI binding sites within the promoters of *Foxj1, Six3, Msi1, Msi2, Pdgfc* and *Myo9a*. Since FOXJ1 is still expressed in *Nfix*^*−/−*^ mice, it is clear that NFIX is not the sole factor driving expression of *Foxj1*. However, the delay in expression of FOXJ1 in *Nfix*^*−/−*^ mice (Fig. 2) indicates that NFIX likely plays an important role in mediating the correct timing of *Foxj1* transcription during ependymal cell maturation. This aberrant timing of *Foxj1* expression may therefore contribute to the failure to maintain a normal ependymal cell layer in these mutant mice. In light of this, we further investigated the capacity of NFIX to activate *Foxj1*-promoter driven activity. Firstly, we used chromatin immunoprecipitation using an anti-NFIX antibody, coupled with quantitative PCR (ChIP-qPCR) to determine whether NFIX bound to the NFI motifs identified within the *Foxj1* promoter (− 935 and − 2856 relative to the *Foxj1* transcription start site). Primers designed to encompass the − 935 and the − 2856 NFI binding motifs revealed enrichment of NFIX binding to these genomic locations (Fig. 3f), indicative of NFIX occupancy at these sites in vivo*.* Enrichment at the − 2856 was more modest that the − 935 site, suggesting that the most proximal site may be more functionally relevant. To test the functional relevance of the proximal site, we used a luciferase assay performed in Neuro2A cells. This assay demonstrated that co-expression of NFIX with a luciferase construct regulated by a fragment of the *Foxj1* promoter containing the − 935 NFI binding motif resulted in significantly more luciferase activity in comparison to an empty vector control (Fig. 3g). At this stage, the functional relevance of the NFI motif at − 2856 remains unclear. Collectively, these data imply that NFIX promotes ependymal cell maturation, at least in part, via the transcriptional activation of the *Foxj1* gene.

The NFI family of transcription factors have been shown to regulate the development of numerous regions of the developing nervous system, including the spinal cord, the cerebellum, the pons and the cerebral cortex [9]. Our findings provide additional evidence highlighting the importance of NFIX in the development of the ependymal cell layer of the lateral ventricles, and hence of the broader ventricular system. One of the phenotypes reported here, namely the delayed differentiation of radial-glial derived cells, that ultimately resulting in more differentiated cells, resembles the previously described phenotype in the hippocampus of these mice [10], suggesting a common mechanism for this factor in promoting normal progenitor cell differentiation. Looking forwards, work such as this that aims to decipher the cellular and molecular mechanisms through which this transcription factor family regulates development will be of critical importance to understanding the aetiology of human disorders. For example, patients haploinsifficient for *NFIA* have been reported to exhibit ventriculomegaly and hydrocephalus [19], but the causes underlying this remain unknown. Given that NFIA is also strongly expressed by ependymal cells [4], and that the NFI family share a common DNA binding motif [21], it is likely that aberrant ependymal cell biology may contribute to the ventricular phenotype of these patients. Similarly, patients haploinsufficient for *NFIX* also exhibit ventricular dilation [32]. Future work will be needed to fully understand the role of NFIX in promoting ependymal cell maturation. For instance, closer investigation of adherens junctional integrity in the absence of *Nfix* is warranted. Analysis of the localisation of adherens junctional components, including E-cadherin, β-catenin and p120, to the subapical membrane of ependymal cells would be one way in which to investigate this. Furthermore, an in-depth study of ciliary development, perhaps via electron microscopy, coupled with in vitro and ex vivo culture systems aimed at assessing ciliary function [1] would provide important insights into the functionality of ependymal cilia in *Nfix*^*−/−*^ mice. More broadly, the use of a conditional *Nfix* allele [25], coupled with an ependymal-specific cre driver such as a *Six3 CreER*^*T2:GFP*^ line will enable the role of NFIX specifically in these cells to be parsed from its role in radial glia and different mature cell types. When used in conjunction with non-biased approaches such as chromatin immunoprecipitation and sequencing (ChIP-seq) and single-cell RNA sequencing (RNA-seq), a global view of the role played by NFIX in ependymal cells will emerge, which will clarify how NFIX regulates ependymal cell biology in both development and in disease.

## Additional files

Additional file 1: Lack of NFIX immunoreactivity in ependymal cells within postnatal *Nfix*^*−/−*^ mice. Coronal sections of wild-type (A-C) and *Nfix*^*−/−*^ (D-F) brains at P15 showing expression of vimentin and NFIX. In wild-type mice, vimentin labelled cells lining the lateral ventricles (LV) clearly expressed NFIX (arrowheads in A-C). In *Nfix*^*−/−*^ mice, however, NFIX expression was absent in ependymal cells (arrows in D and F). Scale bar (in A): 30 μm. (TIFF 7739 kb)

Additional file 2: Abnormal ependymal phenotypes of *Nfix*^*−/−*^ mice. Coronal sections of wild-type (A, C) and *Nfix*^*−/−*^ (B, D-F) brains at P5 (A, B) and P15 (C-F) showing expression of vimentin (green). DAPI labelling is shown in white. The boxed regions in A-F are shown at higher magnification in A’-F’ respectively. At P5 in both the wild-type and the mutant, vimentin^+^ cells can be seen lining the walls of the lateral ventricles (arrows in A’, B’). At P15, this is still seen in the wild-type (arrows in C’). In the mutant however, there were regions in which there were some ependymal cells (arrow in D’), adjacent to areas where ependymal cells were not apparent (asterisk in D’). In other regions of the mutant brain, a thickening of the ependymal cell layer was observed (arrowheads in E’), or complete absence of the ependymal cell layer lining the lateral ventricle (LV; F, F’). The double arrowhead in F’ indicates a vimentin^+^ astrocyte. The dashed lines in F and F’ demarcate the ventricular cavity and the brain parenchmya. Scale bar (in A): A-F 100 μm; A’-F’ 30 μm. (TIFF 12606 kb)

## Abbreviations

ChIP-seq: Chromatin immunoprecipitation and sequencing
FOXJ1: Forkhead box protein J1
NFI: Nuclear factor I
NFIX: Nuclear factor IX

## References

[1] Al Omran AJ, Saternos HC, Liu T, Nauli SM, AbouAlaiwi WA (2015). Live imaging of the ependymal cilia in the lateral ventricles of the mouse brain. J Vis Exp.

[2] Arai Y, Deguchi K, Takashima S (1998). Vascular endothelial growth factor in brains with periventricular leukomalacia. Pediatr Neurol.

[3] Campbell CE, Piper M, Plachez C, Yeh YT, Baizer JS, Osinski JM, Litwack ED, Richards LJ, Gronostajski RM (2008). The transcription factor Nfix is essential for normal brain development. BMC Dev Biol.

[4] Chen KS, Harris L, Lim JWC, Harvey TJ, Piper M, Gronostajski RM, Richards LJ, Bunt J (2017). Differential neuronal and glial expression of nuclear factor I proteins in the cerebral cortex of adult mice. J Comp Neurol.

[5] Dixon C, Harvey TJ, Smith AG, Gronostajski RM, Bailey TL, Piper M (2013). Nuclear factor one x regulates bobby sox during development of the mouse forebrain. Cell and Molec Neurobiol.

[6] Gonzalez AM, Berry M, Maher PA, Logan A, Baird A (1995). A comprehensive analysis of the distribution of FGF-2 and FGFR1 in the rat brain. Brain Res.

[7] Gopalan SM, Wilczynska KM, Konik BS, Bryan L, Kordula T (2006). Nuclear factor-1-X regulates astrocyte-specific expression of the alpha1-antichymotrypsin and glial fibrillary acidic protein genes. J Biol Chem.

[8] Gotz M, Huttner WB (2005). The cell biology of neurogenesis. Nat Rev Mol Cell Bio.

[9] Harris L, Genovesi LA, Gronostajski RM, Wainwright BJ, Piper M (2015). Nuclear factor one transcription factors: divergent functions in developmental versus adult stem cell populations. Dev Dyn.

[10] Harris L, Zalucki O, Gobius I, McDonald H, Osinki J, Harvey TJ, Essebier A, Vidovic D, Gladwyn-Ng I, Burne TH (2016). Transcriptional regulation of intermediate progenitor cell generation during hippocampal development. Development.

[11] Harris L, Zalucki O, Piper M, Heng JI (2016). Insights into the biology and therapeutic applications of neural stem cells. Stem Cells Int.

[12] Heng YH, McLeay RC, Harvey TJ, Smith AG, Barry G, Cato K, Plachez C, Little E, Mason S, Dixon C (2014). NFIX regulates neural progenitor cell differentiation during hippocampal morphogenesis. Cereb Cortex.

[13] Heng YH, Zhou B, Harris L, Harvey T, Smith A, Horne E, Martynoga B, Andersen J, Achimastou A, Cato K (2015). NFIX regulates proliferation and migration within the murine SVZ neurogenic niche. Cereb Cortex.

[14] Jacquet BV, Salinas-Mondragon R, Liang H, Therit B, Buie JD, Dykstra M, Campbell K, Ostrowski LE, Brody SL, Ghashghaei HT (2009). FoxJ1-dependent gene expression is required for differentiation of radial glia into ependymal cells and a subset of astrocytes in the postnatal brain. Development.

[15] Jimenez AJ, Dominguez-Pinos MD, Guerra MM, Fernandez-Llebrez P, Perez-Figares JM (2014). Structure and function of the ependymal barrier and diseases associated with ependyma disruption. Tissue Barriers.

[16] Kokovay E, Goderie S, Wang Y, Lotz S, Lin G, Sun Y, Roysam B, Shen Q, Temple S (2010). Adult SVZ lineage cells home to and leave the vascular niche via differential responses to SDF1/CXCR4 signaling. Cell Stem Cell.

[17] Lavado A, Oliver G (2011). Six3 is required for ependymal cell maturation. Development.

[18] Lim DA, Tramontin AD, Trevejo JM, Herrera DG, Garcia-Verdugo JM, Alvarez-Buylla A (2000). Noggin antagonizes BMP signaling to create a niche for adult neurogenesis. Neuron.

[19] Lu W, Quintero-Rivera F, Fan Y, Alkuraya FS, Donovan DJ, Xi Q, Turbe-Doan A, Li QG, Campbell CG, Shanske AL (2007). NFIA haploinsufficiency is associated with a CNS malformation syndrome and urinary tract defects. PLoS Genet.

[20] Martynoga B, Mateo JL, Zhou B, Andersen J, Achimastou A, Urban N, van den Berg D, Georgopoulou D, Hadjur S, Wittbrodt J (2013). Epigenomic enhancer annotation reveals a key role for NFIX in neural stem cell quiescence. Genes Dev.

[21] Mason S, Piper M, Gronostajski RM, Richards LJ (2009). Nuclear factor one transcription factors in CNS development. Mol Neurobiol.

[22] Matsuda T, Cepko CL (2004). Electroporation and RNA interference in the rodent retina in vivo and in vitro. Proc Natl Acad Sci U S A.

[23] Matuzelski E, Bunt J, Harkins D, Lim JWC, Gronostajski RM, Richards LJ, Harris L, Piper M (2017). Transcriptional regulation of Nfix by NFIB drives astrocytic maturation within the developing spinal cord. Dev Biol.

[24] McAllister JP (2012). Pathophysiology of congenital and neonatal hydrocephalus. Seminars in Fetal & Neonatal Med.

[25] Messina G, Biressi S, Monteverde S, Magli A, Cassano M, Perani L, Roncaglia E, Tagliafico E, Starnes L, Campbell CE (2010). Nfix regulates fetal-specific transcription in developing skeletal muscle. Cell.

[26] Oishi S, Premarathne S, Harvey TJ, Iyer S, Dixon C, Alexander S, Burne TH, Wood SA, Piper M (2016). Usp9x-deficiency disrupts the morphological development of the postnatal hippocampal dentate gyrus. Sci Rep.

[27] Oliver C, Gonzalez CA, Alvial G, Flores CA, Rodriguez EM, Batiz LF (2013). Disruption of CDH2/N-cadherin-based adherens junctions leads to apoptosis of ependymal cells and denudation of brain ventricular walls. J Neuropath and Exp Neurol.

[28] Piper M, Barry G, Harvey TJ, McLeay R, Smith AG, Harris L, Mason S, Stringer BW, Day BW, Wray NR (2014). NFIB-mediated repression of the epigenetic factor Ezh2 regulates cortical development. J Neurosci.

[29] Piper M, Harris L, Barry G, Heng YH, Plachez C, Gronostajski RM, Richards LJ (2011). Nuclear factor one X regulates the development of multiple cellular populations in the postnatal cerebellum. J Comp Neurol.

[30] Pjanic M, Pjanic P, Schmid C, Ambrosini G, Gaussin A, Plasari G, Mazza C, Bucher P, Mermod N (2011). Nuclear factor I revealed as family of promoter binding transcription activators. BMC Genomics.

[31] Plachez C, Cato K, McLeay RC, Heng YH, Bailey TL, Gronostasjki RM, Richards LJ, Puche AC, Piper M (2012). Expression of nuclear factor one a and -B in the olfactory bulb. J Comp Neurol.

[32] Priolo M, Grosso E, Mammi C, Labate C, Naretto VG, Vacalebre C, Caridi P, Lagana C (2012). A peculiar mutation in the DNA-binding/dimerization domain of NFIX causes Sotos-like overgrowth syndrome: a new case. Gene.

[33] Ramirez-Castillejo C, Sanchez-Sanchez F, Andreu-Agullo C, Ferron SR, Aroca-Aguilar JD, Sanchez P, Mira H, Escribano J, Farinas I (2006). Pigment epithelium-derived factor is a niche signal for neural stem cell renewal. Nat Neurosci.

[34] Schnitzer J, Franke WW, Schachner M (1981). Immunocytochemical demonstration of vimentin in astrocytes and ependymal cells of developing and adult mouse nervous system. J Cell Biol.

[35] Spassky N, Merkle FT, Flames N, Tramontin AD, Garcia-Verdugo JM, Alvarez-Buylla A (2005). Adult ependymal cells are postmitotic and are derived from radial glial cells during embryogenesis. J Neurosci.

[36] Vidovic D, Harris L, Harvey TJ, Evelyn Heng YH, Smith AG, Osinski J, Hughes J, Thomas P, Gronostajski RM, Bailey TL (2015). Expansion of the lateral ventricles and ependymal deficits underlie the hydrocephalus evident in mice lacking the transcription factor NFIX. Brain Res.

[37] Wang W, Mullikin-Kilpatrick D, Crandall JE, Gronostajski RM, Litwack ED, Kilpatrick DL (2007). Nuclear factor I coordinates multiple phases of cerebellar granule cell development via regulation of cell adhesion molecules. J Neurosci.

[38] Worthington WC, Cathcart RS (1963). Ependymal cilia: distribution and activity in the adult human brain. Science.

[39] Worthington WC, Cathcart RS (1966). Ciliary currents on ependymal surfaces. Ann N Y Acad Sci.

[40] Yu X, Ng CP, Habacher H, Roy S (2008). Foxj1 transcription factors are master regulators of the motile ciliogenic program. Nat Genet.

[41] Zandt BJ, Liu JH, Veruki ML, Hartveit E (2017). AII amacrine cells: quantitative reconstruction and morphometric analysis of electrophysiologically identified cells in live rat retinal slices imaged with multi-photon excitation microscopy. Brain Struct Funct.

