# Supplementary figures and images for: Transcriptional regulation of ependymal cell maturation within the postnatal brain
Source: Neural Dev. 2018 Feb 16;13:2. doi: 10.1186/s13064-018-0099-4 (PMC5816376; doi:10.1186/s13064-018-0099-4)

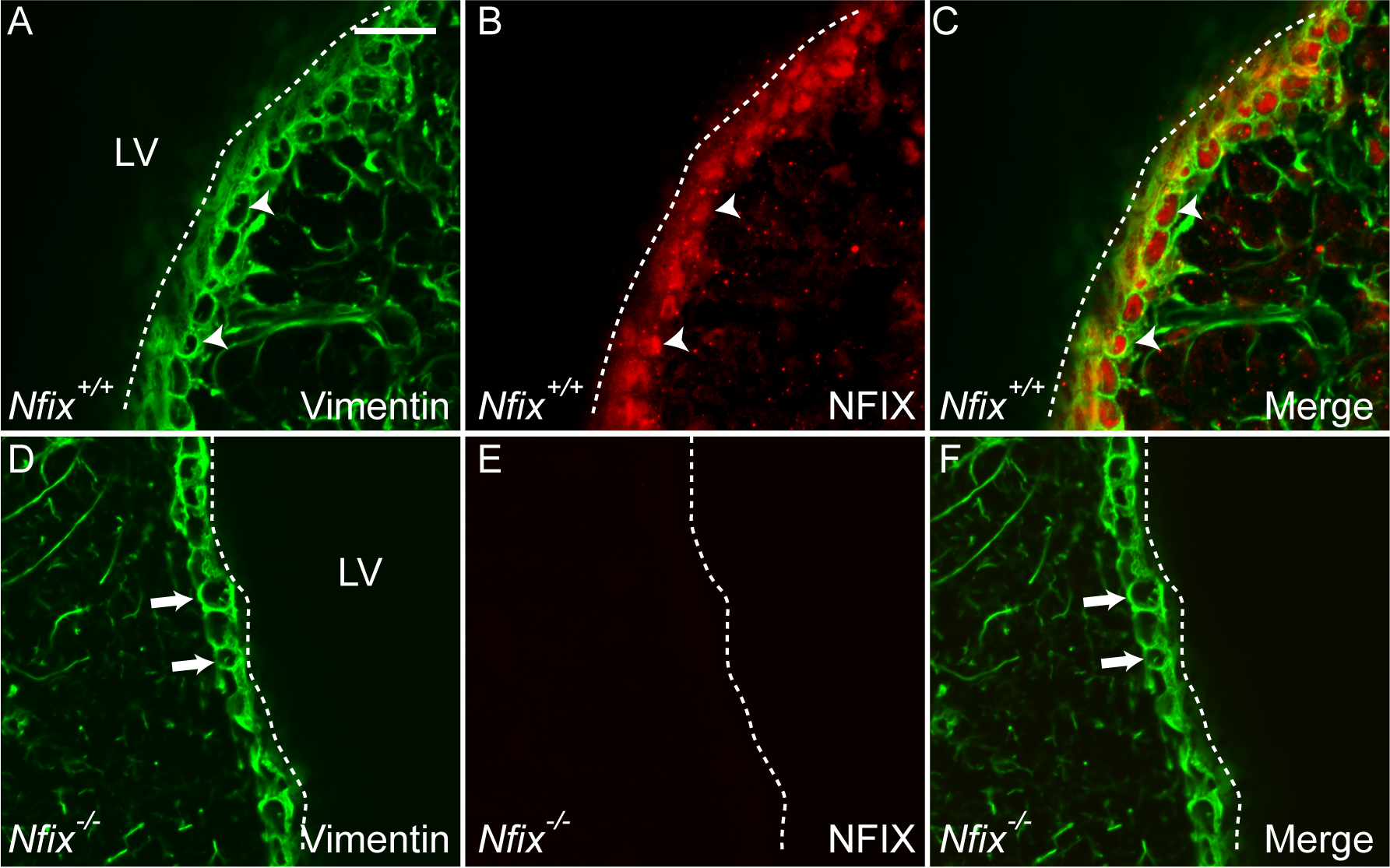

Supplement: Supplementary file 1 — Lack of NFIX immunoreactivity in ependymal cells within postnatal Nfix−/− mice. Coronal sections of wild-type (A-C) and Nfix−/− (D-F) brains at P15 showing expression of vimentin and NFIX. In wild-type mice, vimentin labelled cells lining the lateral ventricles (LV) clearly expressed NFIX (arrowheads in A-C). In Nfix−/− mice, however, NFIX expression was absent in ependymal cells (arrows in D and F). Scale bar (in A): 30 μm. (TIFF 7739 kb) [file 13064_2018_99_MOESM1_ESM.tif]

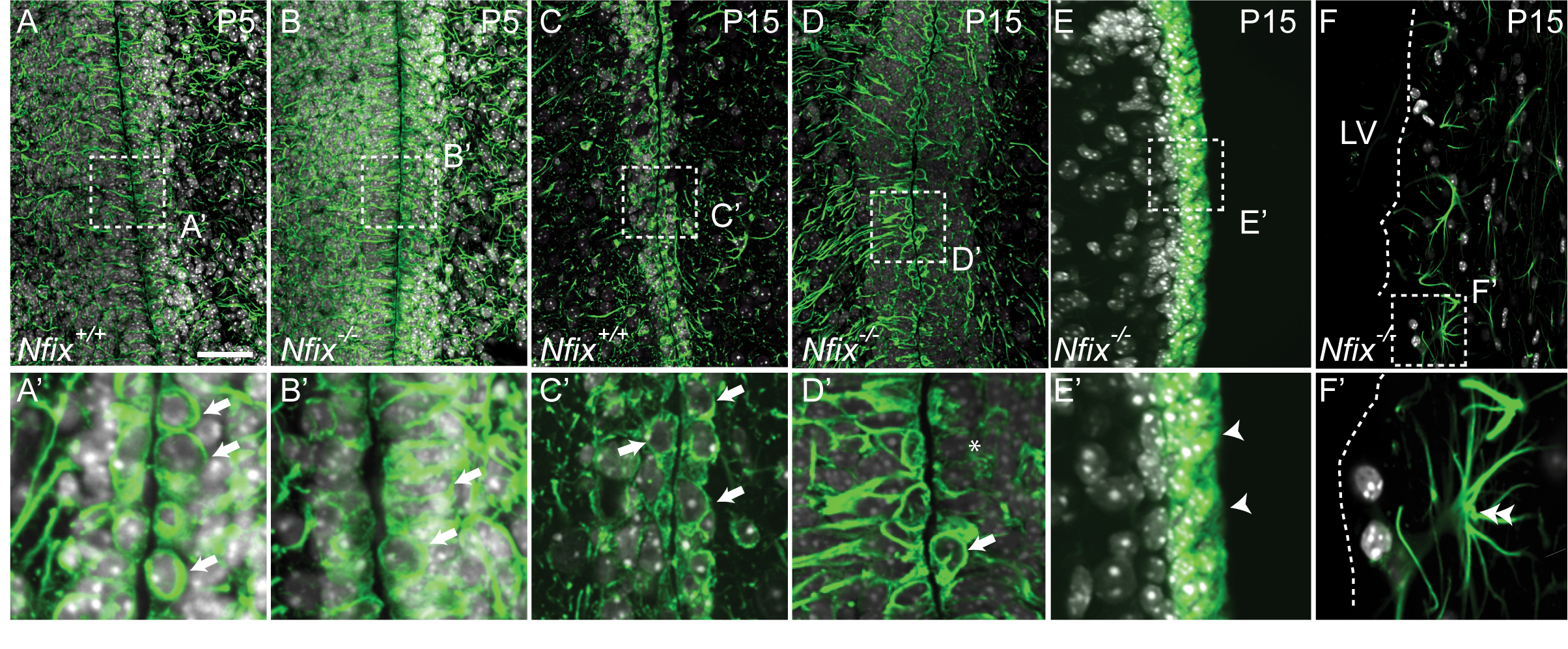

Supplement: Supplementary file 2 — Abnormal ependymal phenotypes of Nfix−/− mice. Coronal sections of wild-type (A, C) and Nfix−/− (B, D-F) brains at P5 (A, B) and P15 (C-F) showing expression of vimentin (green). DAPI labelling is shown in white. The boxed regions in A-F are shown at higher magnification in A’-F’ respectively. At P5 in both the wild-type and the mutant, vimentin+ cells can be seen lining the walls of the lateral ventricles (arrows in A’, B’). At P15, this is still seen in the wild-type (arrows in C’). In the mutant however, there were regions in which there were some ependymal cells (arrow in D’), adjacent to areas where ependymal cells were not apparent (asterisk in D’). In other regions of the mutant brain, a thickening of the ependymal cell layer was observed (arrowheads in E’), or complete absence of the ependymal cell layer lining the lateral ventricle (LV; F, F’). The double arrowhead in F’ indicates a vimentin+ astrocyte. The dashed lines in F and F’ demarcate the ventricular cavity and the brain parenchmya. Scale bar (in A): A-F 100 μm; A’-F’ 30 μm. (TIFF 12606 kb) [file 13064_2018_99_MOESM2_ESM.tif]
